# Alterations in the ability to maintain balance as a result of stochastic resonance whole body vibration in women

**DOI:** 10.1371/journal.pone.0185179

**Published:** 2017-09-22

**Authors:** Karolina Donocik, Magdalena Hartman-Petrycka, Agata Lebiedowska, Barbara Błońska-Fajfrowska

**Affiliations:** Department of Basic Biomedical Science, School of Pharmacy with the Division of Laboratory Medicine, Medical University of Silesia, Katowice, Poland; Tokai University, JAPAN

## Abstract

**Purpose:**

A vertical posture makes it difficult to maintain balance especially in the elderly. Loss of balance leads to falls and injuries. In the present study, we evaluated whether balance maintenance can be improved with the use of stochastic resonance whole body vibration (SR-WBV).

**Methods:**

An examination of balance, involving 187 women aged 19–74 years, was conducted using double-plate posturography pre and post SR-WBV. The SR-WBV trainings were performed using the SRT Zeptor Medical-plus noise device. The entire study lasted 6 weeks, with a total of 12 training sessions, each consisting of nine 45 second series, with a 45 second pause between them.

**Results:**

Post SR-WBV there was a reduction in the value of: the resultant mean velocity (MV) of the movement of COP (centre of pressure) for both lower limbs (B) and in the right lower limb (R) during the test with eyes closed (EC), the mean velocity and mean amplitude (MA) of the movement of COP along the x-axis (ML) of the left lower limb (L) during the test with eyes open (EO) and closed and some additional parameters. Negative correlations between age/index of improvement of MV-EC-B, MV-EC-L and MVML-EC-L, and BMI/index of improvement of MV-EC-B, MVML-EC-B appeared. Height correlated positively with the index of improvement of MV-EC-B and MVML-EC-B.

**Conclusions:**

As a result of SR-WBV, the left leg is more stable along the x-axis and the disproportion between the stability of both legs is reduced. Consequently, body stability is higher. The SR-WBV is more effective in younger, taller and slimmer women. SR-WBV parameters should be optimized so that the training is more beneficial for elderly and shorter women, and for women with a higher BMI.

## Introduction

As a result of evolution, man has reached a vertical posture. Bipedal posture is associated with the release of the upper limbs from their support functions. The remaining support surface is relatively small and limited only to the feet. During daily activities, the centre of gravity moves within and beyond this small base of support, which can cause loss of balance and falls [1]. This problem increases with age and affects 30% of people over 65 years of age and up to 50% after 85 years of age. Injuries from falls are the fifth highest cause of death in the elderly and the highest cause of death among people over 75 years of age [2]. They are the most frequent cause of hospitalization and death among all accidents in Poland. Women are more likely to fall than men. In 2014 more than 49% of hospital stays for men and more than 60% of hospital stays for women were fall-related. The current literature relates primarily to the falls of older people and ignores the problem of the prevalence of falls in younger age groups. Falls among younger people are also common as evidenced by the high frequency of hospitalization for falls in age groups under 65 years old. The real coefficients of fall-related hospitaliszation on 100,000 population were 1,100.1 in the 0–14 age group in 2014, 1,197.6 in the 15–24 age group and 840.7 in the 25–64 age group. In Poland 4,718 people died (the real mortality rate was 12.7 per 100 000 population).as a result of falls in 2014. More than 49% of falls, resulting in fatal injury, are ground level falls caused by slipping or sliding. Falls from stairs and other inclines are rare causes of death (7.3%). For 25% of falls, the circumstances in which the fall occurred are not known [3].

The projection of the centre of gravity is located in a specified area of the support surface, which is approximately 5 cm anterior to the talocrural joint [4]. Even during quiet standing, every activity of the body including heart rate, blood circulation, breathing and tonic activity of the antigravity muscles, causes a slight imbalance, and thus shifts the centre of gravity of the body. Maintaining a stable standing posture involves constantly restoring the centre of gravity of the body to its original position [5].

Posturography indirectly determines the efficiency of the balance system, especially the functions of the nervous and muscular systems [6]. The posturography device captures the movement of the centre of gravity, and at the same time, calculates the point of application of the resultant ground reaction force, known as the centre of foot pressure (*centre of pressure*, COP). Ocetkiewicz et al. [7] showed that the posturography test is a good method to assess balance with highly repeatable measurements. The single plate posturography device records the resultant of pressure in three or four points of the platform while the examined person has both feet placed on one surface. Double-plate posturography device carries out measurements for the lower limbs separately. It allows for the analysis of the phenomenon of stability and symmetry of lower limb loading in the examined person [8]. Determining a less stable limb can make rehabilitation more directed and allow for a very accurate assessment of the effects of exercises on postural stability parameters, such as stochastic resonance whole body vibration (SR-WBV) training.

An accurate analysis of the movement of the centre of gravity of the body, in actual fact its projection, gives an indication of those people with a high risk of recurrent falls. Depending on the posturography platform and software used, various parameters of the movement of the centre of gravity of the body are calculated. Moghadam et al. [9], Pajala et al. [10] and Swanenburg et al. [11] confirmed that the mean amplitude along the x-axis, mean velocity and mean velocity along the x-axis have the greatest predictive value for identifying people with a higher risk of falls.

It is well known that exercise improves stability [12]. However, 32% of adult Poles never perform physical activity, for 7% of Polish adults physical activity occurs less than once a month and another 8% do exercise only 1 to 3 times a month. In consequence, 47% of Poles do not profit sufficiently from the benefits of movement, which may cause a reduction in the ability to maintain balance [13]. Lack of time (64%), fatigue (47%) and lack of mobilization / laziness (39%) are the three most frequently mentioned obstacles to physical activity among adult Poles working in offices [14]. Different devices are being designed in order to obtain a positive effect on the body without performing exercises. One of these is the SRT Zeptor Medical-plus noise device (Zeptoring Deutschland GmbH, Berlin, Germany) which generates stochastic resonance whole-body vibrations. The SR-WBV training sessions on the muscle imbalance program are short. They last about 13 minutes and do not cause fatigue. This results in a positive feedback from participants. In this work we undertook the assessment of whether such a short vibration has a positive impact on balance and can support the physical activity.

Stochastic resonance (SR) occurs in dynamic, nonlinear systems, and is formed by the superimposition of signals from at least two sources. These two sources are generally one signal with a constant value, and a second, variable signal, also called stochastic noise. In the case of an imposition of both signals, specific gain occurs, so sub-threshold impulses can achieve supra-threshold values [15]. Stochastic resonance vibrations interact with variable parameters of the nervous system and create a resonant reaction. This enhanced reaction results in the need for a slight intensity of stimulation to induce neuromuscular activity, while the sinusoidal vibrations of the same strength remain as the subliminal stimulus [16]. Rogan et al. [17] postulated that stochastic resonance dynamics have been found in a selection of anatomical structures such as human spindles [18]. In contrast to a sinusoidal signal, the stochastic resonance stimulus has the ability to change the threshold values of the cell membrane potential [19], which may explain why cells are easily excited under such stochastic conditions [20]. The slight intensity of stimulation allows SR trainings to be performed for the elderly and disabled. In order to generate stochastic resonance whole-body vibrations, double-plate platforms are used [21]. The areas of stochastic resonance application are for persons with balance disability and falls history [17, 22, 23] and for persons of de-conditioning such as post-injury conditions, osteoporosis.

Balance disorders, and the consequences of falls are a social problem, particularly for the aging population in developed countries. Consequently, the evaluation of SR-WBV to maintain the stability of women's bodies including factors such as age, height, and BMI, was the aim of this study.

## Materials and methods

### Recruitment of training participants

**Fig 1:**
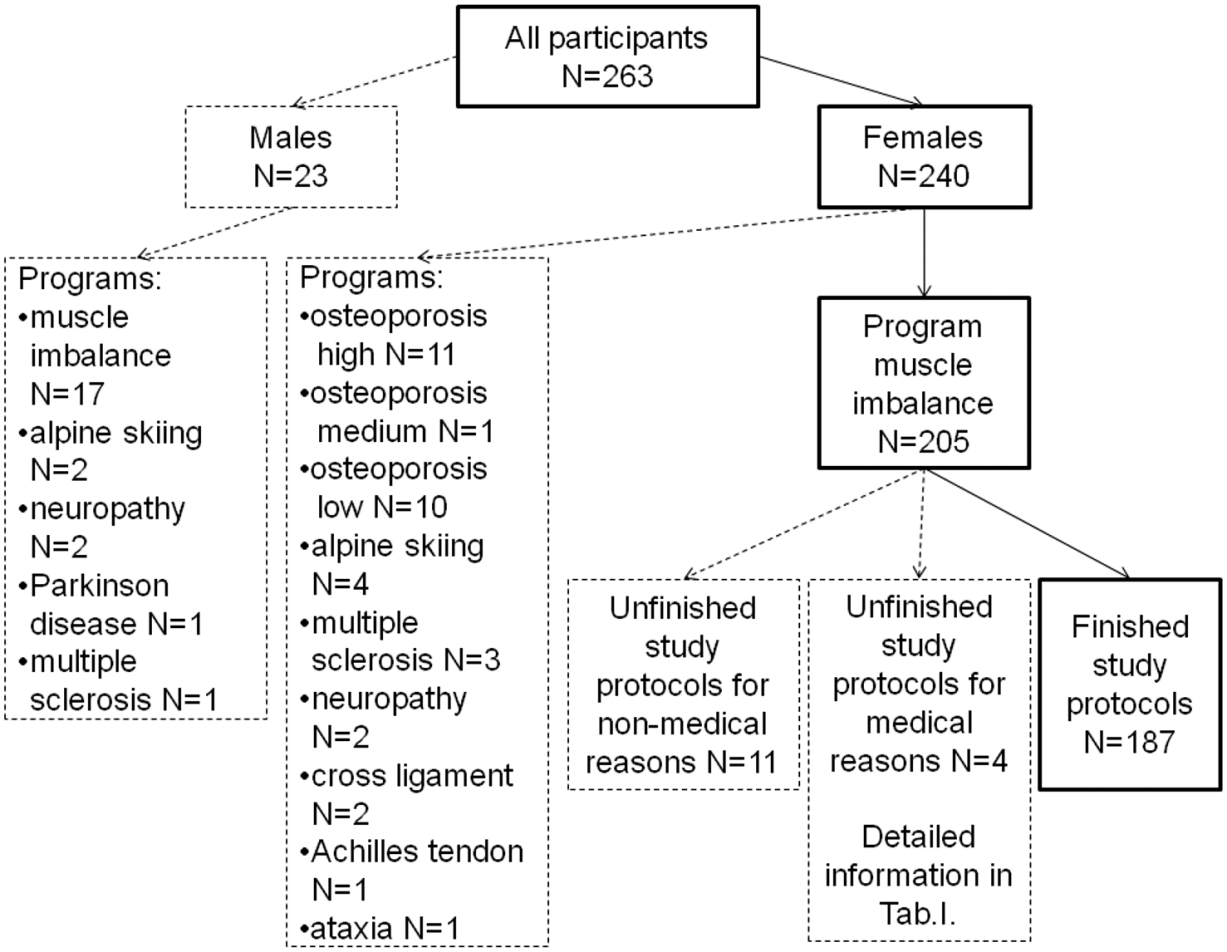
Recruited participants. The broken line indicates people not included in the results of this study.

The study results for the ability to maintain balance described in this paper are part of the results obtained during the wider scientific program studying the effects of stochastic resonance whole body vibration on the human body. This program was implemented in the Medical University of Silesia (SUM) in Katowice, Poland, in 2013–2016. All program participants lived in the Silesia region. The whole region, and especially the Upper Silesian Industrial Region, where the University is located, comprises urbanized and industrialized areas, and, therefore, the majority of the participants are residents of large and medium-sized towns/cities. Information about the project was disseminated among staff, students and attendees of the University of the Third Age at the Faculty of Pharmacy with the Department of Laboratory Medicine, SUM as well as among their families and friends. During the dissemination process, information about the project was provided, such as the most important health contraindications. People with severe health problems did not apply to participate in the project, so it is difficult to estimate the initial number of people informed and willing to take part in the vibration training. Anyone who declared that they were not in good health was examined by a General Practitioner (GP) before the training, and in case of doubt, was asked to provide consent for the vibration training from the specialist who was treating them. Eventually 263 people were qualified for the program. The group was very diverse, both men and women of all ages and with different health conditions. The program of vibration was chosen individually depending on the state of health, from the list of programs offered by the device manufacturer. As a result, 10 different programs were used in the project with varying degrees of vibration intensity. The group that was the most numerous, with very good or good health, sex homogeneous (women) and with the same applied vibration program (muscle imbalance) (Fig 1) was selected for this paper. The muscle imbalance program is characterized by the highest frequency and, among the programs dedicated to rehabilitation, is the one which participants sense most strongly.

5% (N = 11) of women did not complete SR-WBV trainings for non-medical reasons, and 2% (N = 4) of women for medical reasons. The health problems causing the discontinuation of the SR-WBV training in the group selected for the analysis are presented in Table 1.

**Table 1:** The health reasons for quitting the SR-WBV training in the "muscle imbalance" program.

No.	Number of sessions /Level	Age	Health problem in medical history	Reason for quitting the training
**1**	1/medium, 1/high	52	no medical history of health problems	back pain
**2**	1/medium	52	surgery for hernia and vermiform appendix	hyperthyroidism
**3**	4/low	74	overweight, knee pain when climbing stairs, arterial hypertension treated pharmacologically, heart arrhythmia	heart arrhythmia (hospitalisation)
**4**	3/low	62	class II obesity	knee pain, dizziness

Results from SR-WBV training performed in other programs will be calculated after data from sufficient numbers of participants has been collected for statistical analysis.

The results contained in this paper describe the effects of the SR-WBV in maintaining balance in 187 women aged 19 to 74 years (mean 29.0 ± 16.2 years). The height of the participants ranged from 148 to 180 cm (mean 165.6 ± 6.3 cm) and BMI ranged from 16.6 to 35.2 (mean 23.1 ± 4.0). A negative correlation was observed between age and height (R = 0.16; p<0.05) and a positive correlation was observed between age and BMI (R = 0,44; p<0,001). 4% (N = 7) of women reported pain in their lower limbs and 5% (N = 10) back pain. These complaints applied to people over 50 years of age.

The research project was consistent with the Declaration of Helsinki and approved by the Bioethical Committee of Medical University of Silesia (approval number: KNW/0022/KB1/49/I/13). All participants signed a consent form to participate in the experiment.

Inclusion criteria were completion of the participation agreement for SR-WBV training using the muscle imbalance program, female sex, right-handed, the ability to move without support, and the ability to stand motionless with eyes closed for one minutes.

Exclusion criteria were acute inflammations, fresh surgical wounds, serious injury, thrombosis, cancer, severe arthritis, advanced discopathy, severe pain especially in knee and hip joints and spine, heart pacemaker, severe arrhythmia, severe coronary heart disease, serious unregulated hypertension, retinal detachment, nausea and malaise, dizziness.

### Double plate posturography

**Fig 2:**
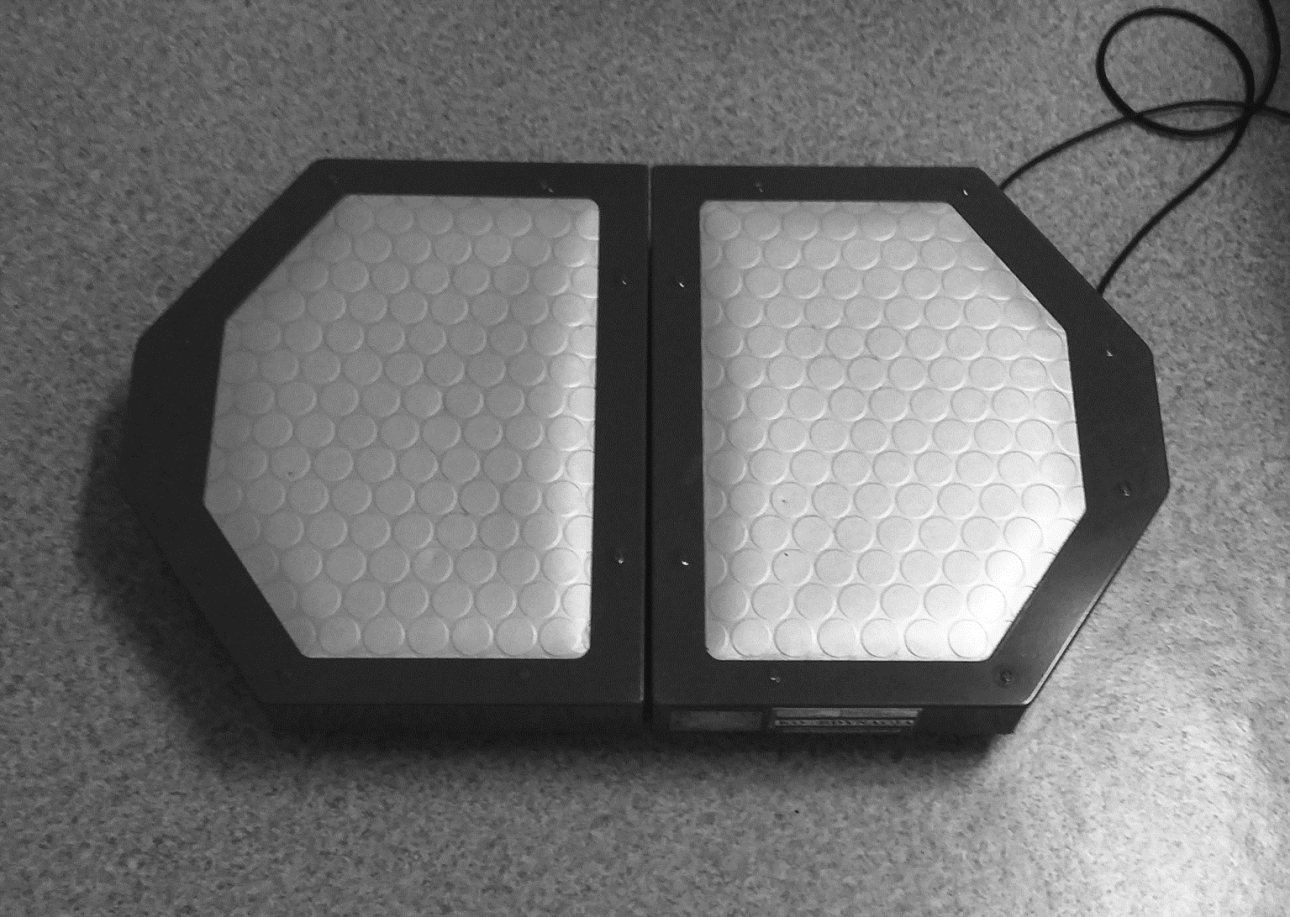
Double plate posturography device.

Both before the first SR-WBV training and again at the end of the entire study, a double plate posturography (Koordynacja, Radom, Poland) (Fig 2) was used to test the participants’ balance after 5 minutes of rest in a sitting position.

During the examination, the subject placed each bare foot on a separate plate on the device, and maintained a relaxed position with the upper limbs along the body. Two attempts were performed, one with eyes open (EO) and one with eyes closed (EC), for 30 seconds each.

The following parameters, describing the movement of the centre of pressure (COP), have been taken into consideration: the sway path (SP) [mm], the sway path along the y-axis (SPAP) [mm] and x-axis (SPML) [mm], the mean amplitude (MA) [mm], the mean amplitude along the y-axis (MAAP) [mm] and the x-axis (MAML) [mm], the mean velocity (MV) [mm/s], the mean velocity along the y-axis (MVAP) [mm/s] and the x-axis (MVML) [mm/s], the sway area (SA) [mm^2^], the mean frequency (MF) [Hz], and the time radius (TR) [%], which is the percentage of time spent by the COP in a circle with a 5 mm radius.

These parameters were calculated independently for the right lower limb (R), left lower limb (L) and as the resultant parameters for both limbs (B).

Disproportion between lower limbs was calculated as a module of different values of posturographic parameters obtained for the left and the right lower limbs (e.g. IR-LI SPML-EC).

The index of improvement was calculated as a difference between the values of the parameters measured before training and the same posturograph parameters measured after SR-WBV training (eg pre SPML-EC—post SPML-EC).

Based on the results presented by Moghadam et al. [9], Pajala et al. [10] and Swanenburg et al. [11], the most important posturographic parameters are: MV, MVML and MAML. Additional parameters were also analyzed.

### SR-WBV training

**Fig 3:**
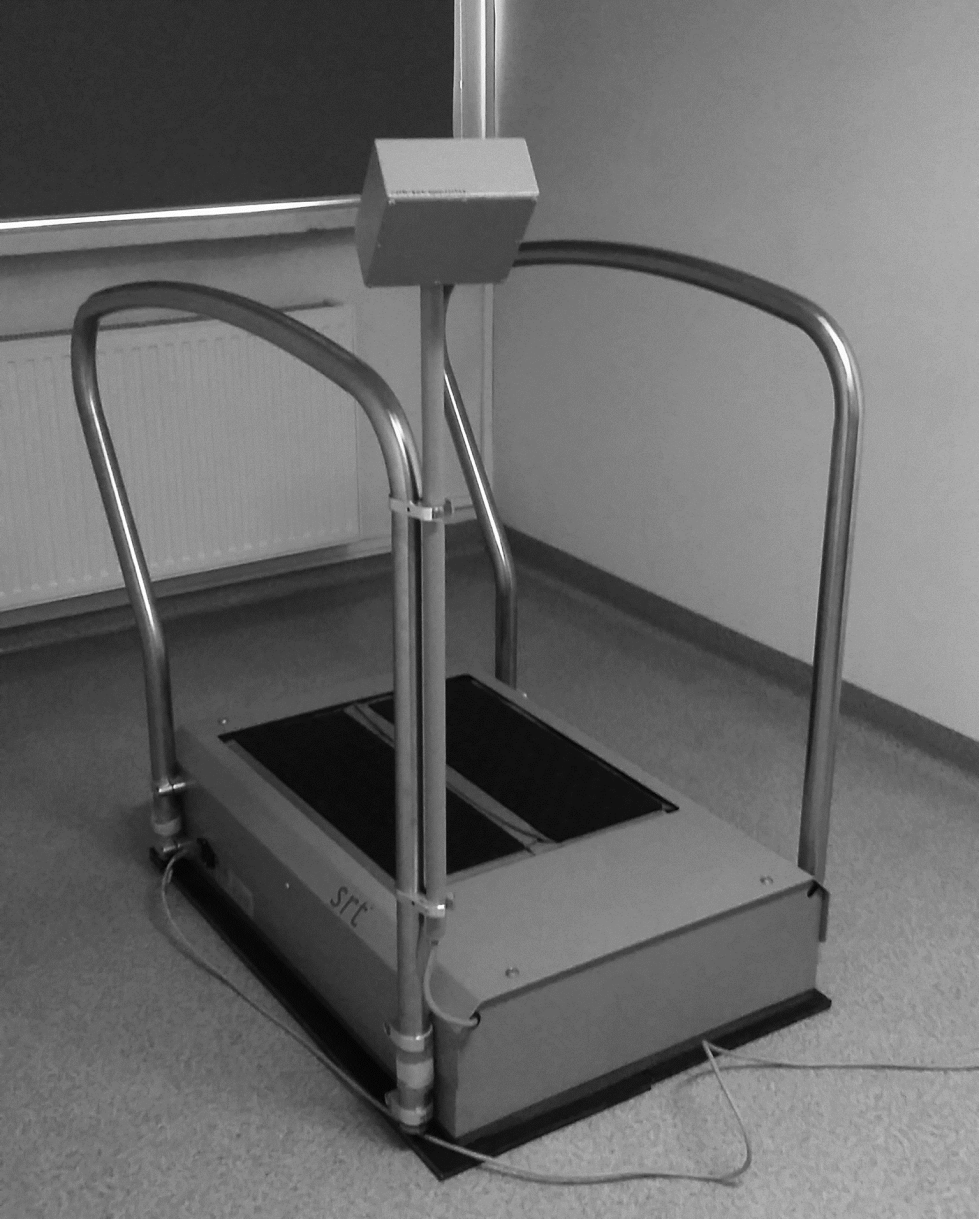
SRT Zeptor Medical-plus noise device.

The SR-WBV training was performed using the SRT Zeptor Medical-plus noise device (Zeptoring Deutschland GmbH, Berlin, Germany) (Fig 3).

This device is used in rehabilitation and sports, but its influence on the organism is still studied in various aspects. During training, the patient put both feet on the plates of the device and stood with slightly bent knees. The plate units started to move in a disorganized way, after running the selected program (muscle imbalance). This movement disturbs the balance of the body and at the same time initiates all body mechanisms correcting imbalance. The entire study lasted 6 weeks. Each week, the volunteers participated in two training sessions with at least a one-day break between the sessions.

In women with a health problem in their medical history, vibration training using muscle imbalance program was started with a low intensity (2–6 Hz; 7 series lasting 50 s, with 40 s pause between each). If the women did not report discomfort during or after the next session, training was performed at medium intensity (2–6 Hz, 8 series lasting 60 s, with 60 s pause between each) and then at high intensity (2–8 Hz, 9 series lasting 45 s, with 45 s pause between each). In healthy participants with no medical history of health problems the training was started with one session at medium intensity. A minimum of 10 sessions were performed at high intensity. In all sessions the first and last series were characterized by the lowest vibrations, while the middle ones were the highest. There was also a progressive increase and then a decrease in the intensity of the stimulus in each series.

### Statistical analyses

Disproportion between lower limbs was calculated as a module of different values of posturographic parameters obtained for the left and the right lower limbs (e.g. IR-LI SPML-EC).

The index of improvement was calculated as a difference between the values of the parameters measured before training and the same posturograph parameters measured after SR-WBV training (eg pre SPML-EC—post SPML-EC).

Based on the results presented by Moghadam et al. [9], Pajala et al. [10] and Swanenburg et al. [11], the most important posturographic parameters are: MV, MVML and MAML. Additional parameters were also analyzed.

Statistical analyses were carried out using Microsoft Excel 2007 and Statistica 10.0 software. The Shapiro-Wilk test was used to analyse normality. Homogeneity of variance was assumed using Levene's test. Post-hoc power (PHP) was calculated by α = 0.05. The Student's *t-*test was used to compare posturograph parameters pre- and post- SR- WBV and to assess disproportions between right and left lower limb pre- and post- SR-WBV. All tests were two-tailed. Spearman's rank correlation coefficient was used to evaluate correlations between age, height and BMI, and the index of improvement. Spearman rank correlation values were expressed using r. Significance was set at *p* less than 0.05.

## Results

The older the woman, the lower the height (R = -0.30; p <0.001) and the higher the BMI (R = 0.44; p <0.001).

### Posturographic parameters pre SR-WBV vs post SR-WBV

**Fig 4:**
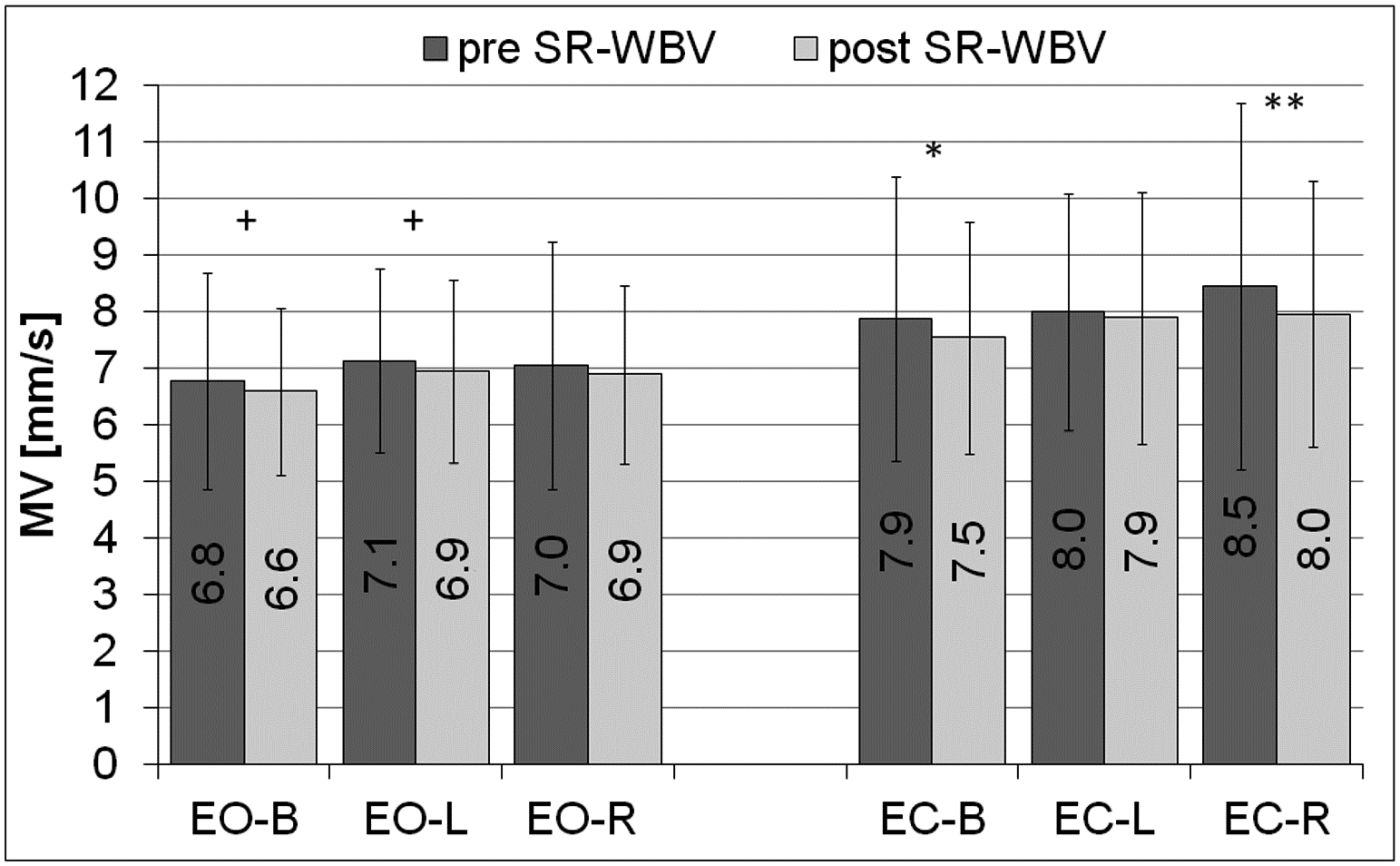
Mean velocity of the movement of COP, pre and post SR-WBV. COP- the centre of pressure, MV- the mean velocity of the movement of COP, EO- the test performed by volunteers with eyes open, EC- the test performed by volunteers with eyes closed, L- the parameters calculated independently for the left lower limb, R- the parameters calculated independently for the right lower limb, B- the resultant parameters calculated for both limbs, pre SR-WBV- the test performed before SR-WBV trainings, post SR-WBV- the test performed after SR-WBV trainings, ^+^p = 0.07, PHP = 0.5; *p<0.05, PHP = 0.6; **p<0.05, PHP = 0.8.

Post SR-WBV a trend in the reduction of the mean velocity (MV) of the movement of COP (the centre of pressure) of the left lower limb (L) (p = 0.07, PHP = 0.5) and the resultant mean velocity (MV) of the movement of COP (the centre of pressure) for both limbs (B) (p = 0.07, PHP = 0.5), was observed during the test with eyes open (EO) compared to the same measurements pre SR-WBV (Fig 4). A value of the mean velocity (MV) of the movement of COP of the right lower limb (R) (p<0.05, PHP = 0.6) and the resultant mean velocity (MV) of the movement of COP for both limbs (B) was significantly lower post SR-WBV during the test with eyes closed (EC) (p<0.05, PHP = 0.8) (Fig 4).

**Fig 5:**
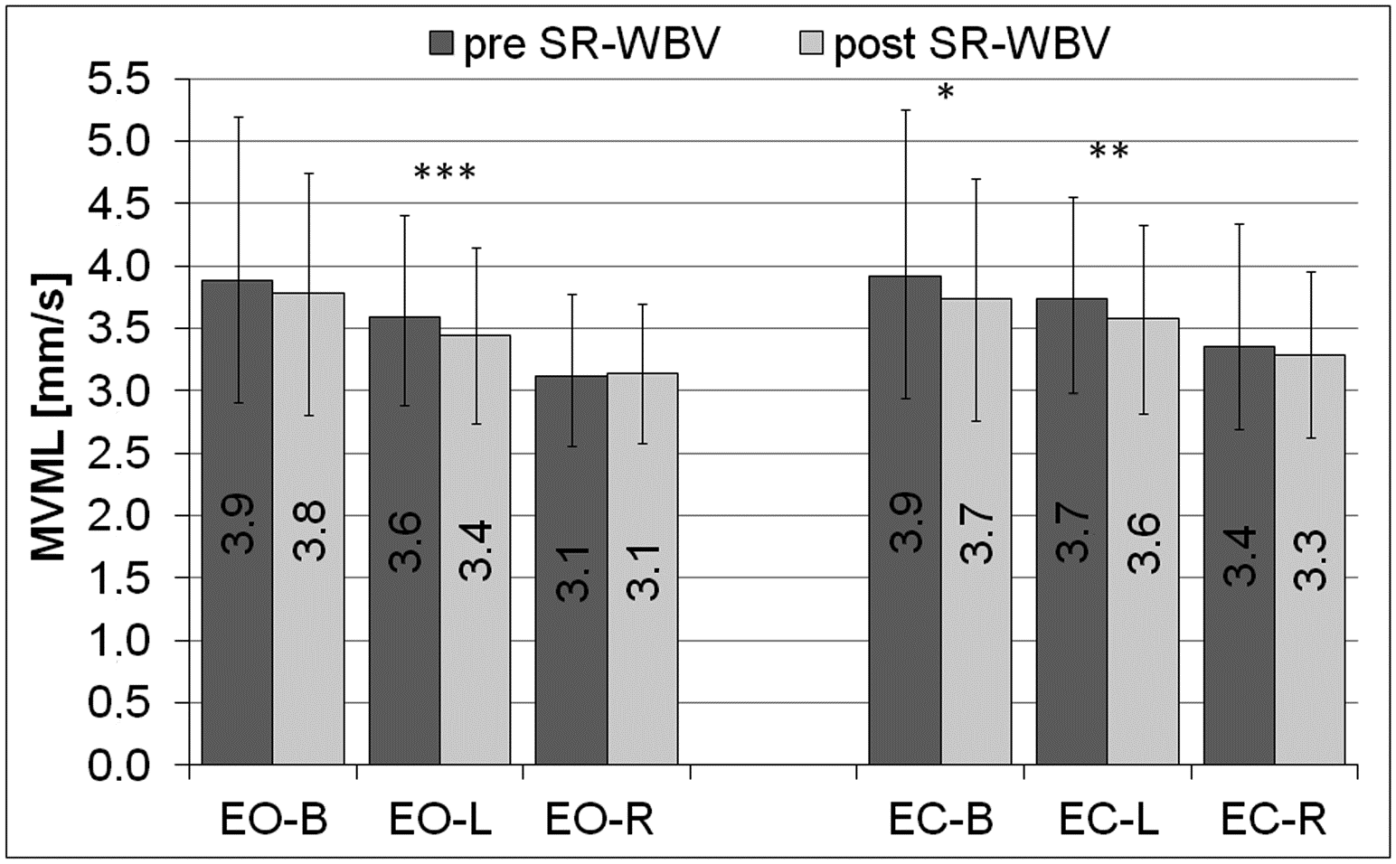
Mean velocity of the movement of COP along the x-axis, pre and post SR-WBV. COP- the centre of pressure, MVML- the mean velocity of the movement of COP along the x-axis, EO- the test performed by volunteers with eyes open, EC- the test performed by volunteers with eyes closed, L- the parameters calculated independently for the left lower limb, R- the parameters calculated independently for the right lower limb, B- the resultant parameters calculated for both limbs, pre SR-WBV- the test performed before SR-WBV trainings, post SR-WBV- the test performed after SR-WBV trainings, *p<0.05, PHP = 0,7; **p<0.01, PHP = 0,9; ***p<0.01, PHP = 0,99.

Post SR-WBV the mean velocity of the movement of COP along the x-axis (MVML) of the left lower limb (L), during the test with eyes open (EO) (p<0.01, PHP = 0.99) and with eyes closed (EC) (p<0.01, PHP = 0.9) was significantly lower compared with the same measurements pre SR-WBV (Ryc.5.) There was also a significant reduction in the resultant mean velocity of the movement of COP along the xaxis (MVML) for both limbs (B) during the test with eyes closed (EC) (p<0.05, PHP = 0.7) (Fig 5).

**Fig 6:**
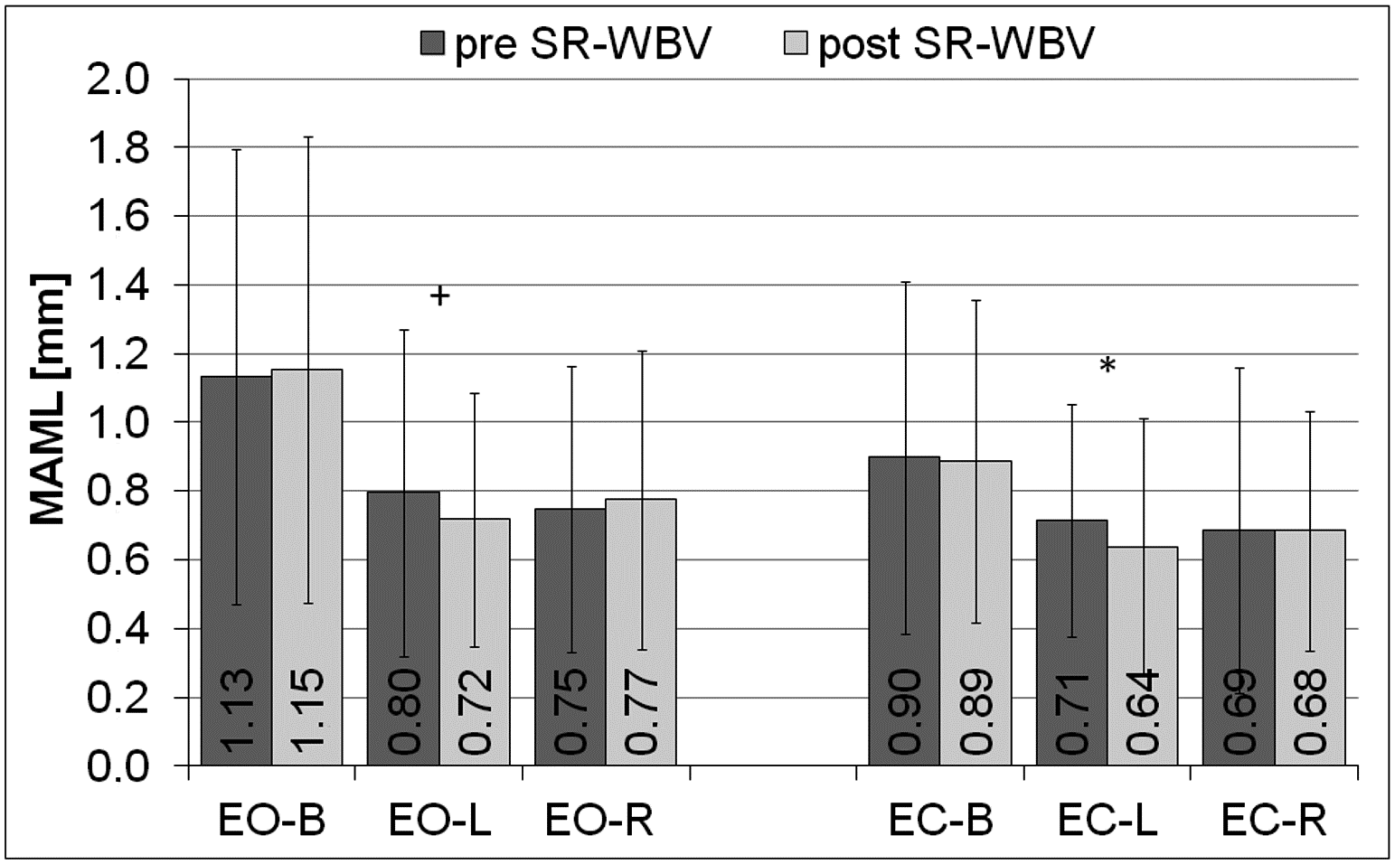
Mean amplitude of the movement of COP along the x-axis, pre and post SR-WBV. COP- the centre of pressure, MAML- the mean amplitude of the movement of COP along the x-axis, EO- the test performed by volunteers with eyes open, EC- the test performed by volunteers with eyes closed, L- the parameters calculated independently for the left lower limb, R- the parameters calculated independently for the right lower limb, B- the resultant parameters calculated for both limbs, pre SR-WBV- the test performed before SR-WBV trainings, post SR-WBV- the test performed after SR-WBV trainings, ^+^p = 0.06, PHP = 0.5; *p<0.05, PHP = 0.8.

The reduction of the mean amplitude along the X-axis (MAML) after SR-WBV training was observed in the left lower limb (L) during both tests: with eyes open (EO) (p = 0.06, PHP = 0.5) and eyes closed (EC) (p<0,05, PHP = 0.8) (Fig 6).

During the test with eyes open (EO) post SR-WBV the trend was a reduction in the resultant value of the sway path (SP) of the movement of COP for both limbs (B) of (p = 0.07, PHP = 0.3) and a significant reduction in the sway path along the x-axis (SPML) of the movement of COP of the left lower limb (L) (p<0.01, PHP = 0.7) compared with values of both parameters pre SR-WBV (S1 Table).

During the test with eyes closed (EC) there was a reduction in many additional parameters after SR-WBV training compared to their values before training: the sway path (SP) (p<0.05, PHP = 0.6), the sway path along the x-axis (SPML) (p<0.05, PHP = 0.7) and the resultant mean frequency (MF) (p<0.05, PHP = 0.6) of the movement of COP for both limbs (B); the sway path along the x-axis (SPML) of the movement of COP of the left lower limb (L) (p<0.01, PHP = 0.9); the sway path (SP) (p<0.05, PHP = 0.7), the sway path along the y-axis (SPAP) (p<0.01, PHP = 0.8) and the mean velocity along the y-axis (MVAP) (p<0.01, PHP = 0.7) of the movement of COP of the right lower limb (R) (S2 Table).

### Disproportion of stability between lower right and lower left limbs pre SR-WBV vs post SR-WBV

**Fig 7:**
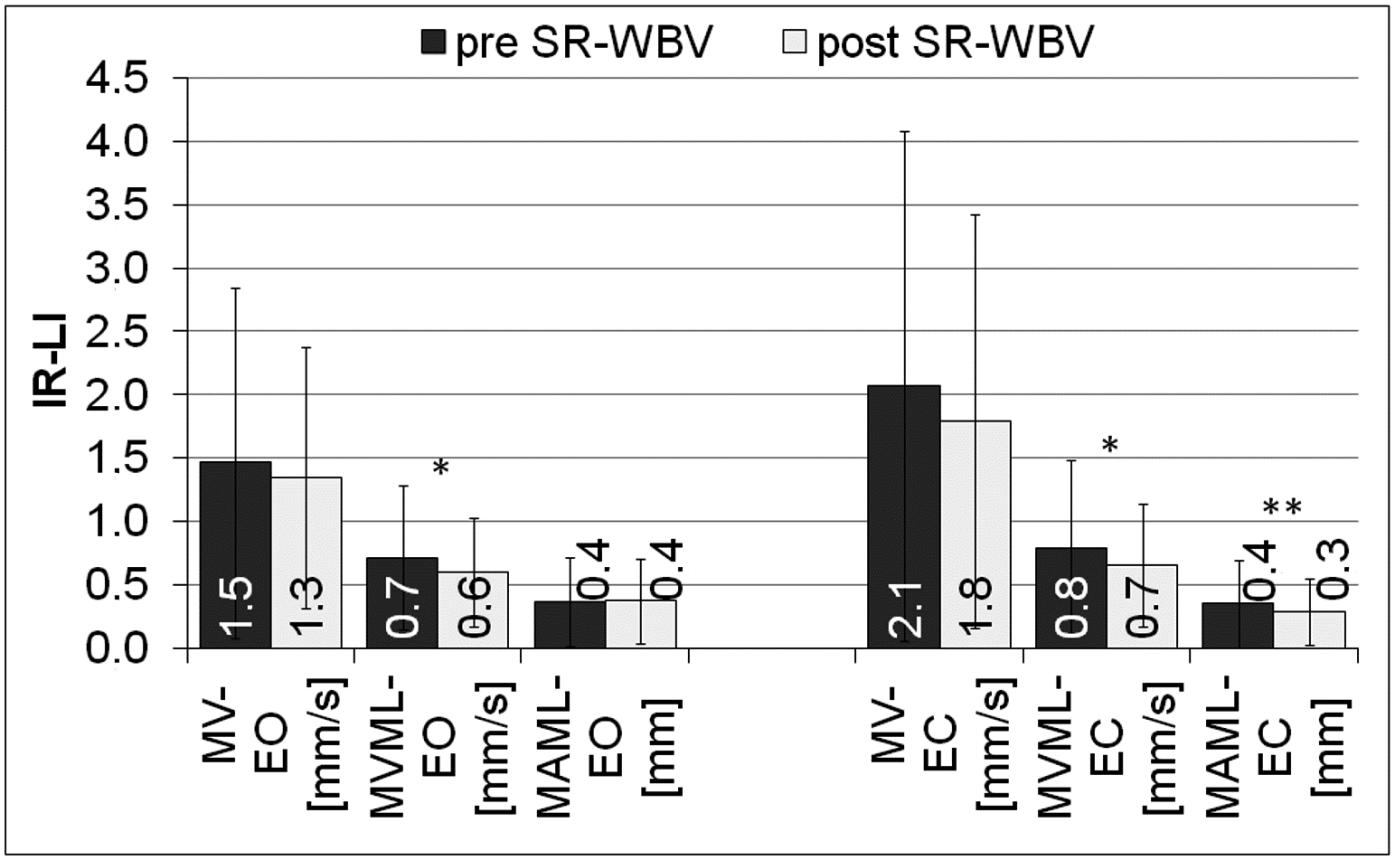
Disproportion of the values of the most important posturographic parameters of lower limbs pre and post SR-WBV. IR-LI—the module of different values obtained for the right and left lower limbs, MV- the mean velocity of the movement of COP, MVML—the mean velocity of the movement of COP along the x-axis, MAML—the mean amplitude of the movement of COP along the x-axis, EO- the test performed by volunteers with eyes open, EC- the test performed by volunteers with eyes closed, pre SR-WBV- the test performed before SR-WBV trainings, post SR-WBV- the test performed after SR-WBV trainings, *p<0.05, PHP = 0.5; **p<0.01, PHP = 0.7.

The reduction of disproportion between lower right and lower left limbs post SR-WBV was observed. The reduction was significant and referred to the mean velocity of the movement of COP along x-axis (MVML) during the test with eyes open (EO) (p<0.05, PHP = 0.5) and eyes closed (EC) (p<0.05, PHP = 0.5) (Fig 7) Post SR-WBV, during the test with eyes closed (EC) the significant reduction of disproportion of the values of mean amplitude of the movement of COP along x-axis (MAML) (p<0.01, PHP = 0.7) was shown (Fig 7).

Among additional parameters the reduction of disproportion between lower right and lower left limbs was observed for the value of the sway path along the x-axis (SPML) during both tests with eyes open (EO) (p<0.05, PHP = 0.7) and eyes closed (EC) (p<0.01, PHP = 0.7) (S3 Table).

### Correlations between age, height, BMI and the index of improvement of posturographic parameters

During the test with eyes open (EO) the correlations between age, height, BMI and the index of improvement of the most important posturographic parameters: the mean velocity (MV), the mean velocity along the x-axis (MVML) and the mean amplitude along the x-axis of the movement of COP measured independently for left (L) and right (R) lower limbs and as the resultant parameters for both limbs (B) were not found (Table 2). After analysing the results of tests performed with participants with eyes closed the situation was different (EC). Age correlated positively with the index of improvement of: the resultant mean velocity (MV) of the movement of COP for both lower limbs (B) (r = -0.23; p<0.01), the mean velocity (MV) of the movement of COP of the left lower limb (L) (r = -0.29; p<0.001) and the mean velocity along the x-axis (MVML) of the movement of COP of the right lower limb (L) (r = -0.29; p<0.001) (Table 2). There was a very weak negative correlation between BMI and the index of improvement of the resultant mean velocity (MV) of the movement of COP for both lower limbs (B) (r = -0.15; p<0.05) and the resultant mean velocity along the x-axis (MVML) of the movement of COP for both limbs (B) (r = -0.16; p<0.05)(Table 2). Moreover, height correlated positively with the index of improvement of the mean velocity (MV) of the movement of COP of the left lower limb (L) (r = 0.23; p<0.01) and the mean velocity along the x-axis (MVML) of the movement of COP of the left lower limb (L) (r = 0.16; p<0.05) (Table 2).

**Table 2:** Correlations between age, height, BMI and the index of improvement of the most important posturographic parameters.

	Parameter	r	p	Parameter	R	p	Parameter	r	p
**Age/ Index of improvement**	MV-EO-B [mm/s]	-0.04	ns	MV-EO-L [mm/s]	-0.11	ns	MV-EO-P [mm/s]	0.06	ns
	MVML-EO-B [mm/s]	0.05	ns	MVML-EO-L [mm/s]	-0.01	ns	MVML-EO-P [mm/s]	-0.04	ns
	MAML-EO-B [mm]	0.13	ns	MAML-EO-L [mm]	-0.06	ns	MAML-EO-P [mm]	0.04	ns
	**MV-EC-B [mm/s]**	**-0.23**	**<0.01**	**MV-EC-L [mm/s]**	**-0.29**	**<0.001**	MV-EC-P [mm/s]	-0.06	ns
	MVML-EC-B [mm/s]	-0.06	ns	**MVML-EC-L [mm/s]**	**-0.19**	**<0.05**	MVML-EC-P [mm/s]	-0.10	ns
	MAML-EC-B [mm]	-0.06	ns	MAML-EC-L [mm]	-0.09	ns	MAML-EC-P [mm]	0.14	ns
**Height/ Index of improvement**	MV-EO-B [mm/s]	0.07	ns	MV-EO-L [mm/s]	0.09	ns	MV-EO-P [mm/s]	0.02	ns
	MVML-EO-B [mm/s]	-0.00	ns	MVML-EO-L [mm/s]	0.03	ns	MVML-EO-P [mm/s]	0.04	ns
	MAML-EO-B [mm]	0.02	ns	MAML-EO-L [mm]	0.12	ns	MAML-EO-P [mm]	-0.06	ns
	MV-EC-B [mm/s]	0.06	ns	**MV-EC-L [mm/s]**	**0.23**	**<0.01**	MV-EC-P [mm/s]	-0.07	ns
	MVML-EC-B [mm/s]	-0.05	ns	**MVML-EC-L [mm/s]**	**0.16**	**<0.05**	MVML-EC-P [mm/s]	-0.07	ns
	MAML-EC-B [mm]	0.01	ns	MAML-EC-L [mm]	0.06	ns	MAML-EC-P [mm]	-0.14	ns
**BMI/ Index of improvement**	MV-EO-B [mm/s]	-0.02	ns	MV-EO-L [mm/s]	-0.00	ns	MV-EO-P [mm/s]	0.03	ns
	MVML-EO-B [mm/s]	-0.04	ns	MVML-EO-L [mm/s]	0.01	ns	MVML-EO-P [mm/s]	-0.05	ns
	MAML-EO-B [mm]	0.03	ns	MAML-EO-L [mm]	-0.05	ns	MAML-EO-P [mm]	0.08	ns
	**MV-EC**-**B [mm/s]**	**-0.15**	**<0.05**	MV-EC-L [mm/s]	-0.11	ns	MV-EC-P [mm/s]	-0.04	ns
	**MVML-EC**-**B [mm/s]**	**-0.16**	**<0.05**	MVML-EC-L [mm/s]	-0.04	ns	MVML-EC-P [mm/s]	-0.04	ns
	MAML-EC-B [mm]	-0.12	ns	MAML-EC-L [mm]	-0.03	ns	MAML-EC-P [mm]	0.10	ns

MV—the mean velocity, MVML—the mean velocity along the x-axis, MAML—the mean amplitude along the x-axis, EO- the test performed by volunteers with eyes open, EC- the test performed by volunteers with eyes closed, L- the parameters calculated independently for the left lower limb, R- the parameters calculated independently for the right lower limb B- the resultant parameters calculated for both limbs, r = rank correlation values, p—significance, ns—not significant.

There were no significant correlations between age, height, BMI and the index of improvement of the additional posturographic parameters: the sway path (SP), the sway path along the y-axis (SPAP), the sway path along the x-axis (SPML), the mean amplitude (MA), the mean amplitude along the y-axis (MAAP), the mean velocity along the y-axis (MVAP), the sway area (SA), the mean frequency (MF), the time radius (TR) of COP measured independently for left (L) and right (R) lower limbs and as the resultant parameters for both lower limbs (B) (S4 Table).

During the tests with eyes closed (EC) post SR-WBV multiple correlations between age, height, BMI and the index of improvement of additional posturographic parameters were observed (S5 Table). The age correlated negatively with the index of improvement of: the sway path (SP) of the movement of COP of the left lower limb (L) (r = -0.29) and the resultant sway path (SP) of the movement of COP for both limbs (B) (r = -0.23), the sway path along the y-axis (SPAP) of the movement of COP of the left lower limb (L) (r = -0.29) and the resultant sway path along the y-axis (SPAP) of the movement of COP for both limbs (B) (r = -0.24), the sway path along the x-axis (SPML) of the movement of COP of the left lower limb (L) (r = -0.19), the mean velocity along the y-axis (MVAP) of the movement of COP of the left lower limb (L) (r = -0.29) and the resultant mean velocity along the y-axis (MVAP) of the movement of COP for both limbs (B) (r = -0.24) along with the sway area (SA) of the movement of COP of the left lower limb (L) (r = -0.17). The negative correlations between the women’s BMI and the index of improvement of the sway path (SP) (r = -0.16) and the resultant sway path along the x-axis (SPML) (r = -0.16) of the movement of COP for both limbs were shown (B). The height correlated positively with the sway path (SP) (r = 0.23), the sway path along the y-axis (SPAP) (r = 0.20) and the sway path along the x-axis (SPML) (r = 0.15) and the mean velocity along the y-axis (MVAP) (r = 0.20) of the movement of COP of the left lower limb (L).

## Discussion

The SR-WBV training was well received by most of the women because it did not cause fatigue, except for some minor delayed muscle soreness in thighs or legs. The full cycle of mechanical vibrations with stochastic resonance improved body stability by reducing the mean velocity (MV) and the mean amplitude (MA) of the movement of COP along the x-axis (ML) of left lower limb (L) during both tests: with eyes open (EO) and eyes closed (EC). The values of resultant mean velocity of the movement of COP (MV) for both limbs (B) and the mean velocity of the movement of COP of the left lower limb (L) during tests with eyes open (EO) and the resultant mean velocity of the movement of COP (MV) for both limbs (B) and the mean velocity of the movement of COP of the right lower limb (R) during tests with eyes closed (EC) were lower. The values of some of the additional posturographic parameters mentioned in this study were also lower.

Stability improvements post SR-WBV stimulation were also emphasized as a reduction of disproportions between lower right and lower left limbs. It referred to MVML (the mean velocity of the movement of COP along the x-axis) parameters during tests, with eyes in both open and closed states and to MAML (the mean amplitude of COP movement of the movement of COP along the x-axis) parameters during tests with eyes closed.

The results of this study are consistent with the other scientific results. Rogan at al. [24], could found that with SR-WBV with 6 Hz, noise level 4 shows benefit improvements in frail elderly individuals on tests: Short Physical Performance Battery—SPPB (ES 0.52), Expanded Timed Get Up-and-Go—ETGUG (part sit-to-stand movement: ES 0.81; total time: ES 0.85) and chair rising—CR (ES 0.66). Dittrich et al. [25] demonstrated an increase in muscle strength, and improvement in motor function and balance in patients older than 60 years of age. In that study, volunteers were exposed to SR-WBV at a frequency of 12.5 Hz, at a rate of 3 times a week, for 3 months. In another study, Patients with polyneuropathy were exposed to SR-WBV 2 times a week for 8 weeks. Each session consisted of five 1-minute cycles of vibration at a frequency of 6 Hz. The data analysis showed a significant reduction of pain and an improvement in stability of the respondents, especially on the X axis [26]. The positive effects of SR-WBV on the human body posture have also been reported by Turbanski et al. [27]. Patients with Parkinson's disease were exposed to SR-WBV at a frequency of 6 Hz, for 5 series of 1 minute each. Priplata et al. [28, 29, 30] used vibrating insoles to generate stochastic resonance vibrations, and reported an improvement in stability after application. Recent Elfering at al. [31, 32] research results have also shown the usefulness of SR-WBV. It includes SR-WBV as an option in the primary prevention of balance-related injury [32], and in musculoskeletal complaints and falls [31]. The researchers investigated the influence of SR-WBV training among Swiss employees. Elfering at al. [33] also conducted research investigating the acute effects of verum SR-WBV (6 Hz, noise level 4) vs. sham SR-WBV (2 Hz, noise level 0) and proved the increase of musculoskeletal relaxation and capacity immediately after verum SR-WBV.

The effectiveness of SR-WBV has not been confirmed in every study. Gaßner et al. [34] divided patients with Parkinson's disease into two groups. In the first group, patients were treated with SR-WBV at a frequency of 6 Hz, 2–3 times a week for 5 weeks. Each session consisted of 5 one-minute sessions. In the second group, control patients were standing on a platform in a same position but without vibrations. Gaßner et al. found a positive effect of vibrations, although similar to the effect achieved in the control group. In 2015, Rogan et al. [35] published their studies in which they estimated the influence of SR-WBV on postural static and dynamic stability among the elderly. The study lasted 4 weeks with a frequency of 5 Hz. Each session consisted of 5 one-minute vibrations. The data analysis did not show any significant changes in volunteers’ postural stability measured by posturography, but researchers admitted that the study was too short and the frequencies used were too low.

The structures of the referenced surveys were different from those in our study. In our study, volunteers were exposed to stochastic vibrations twice a week for 6 weeks. Each cycle consisted of nine series each consisting of 45 seconds of vibration at a frequency of 2–8 Hz, with 45 second breaks between them.

Based on the reports mentioned above and the results described in this paper, it can be assumed that a vibration effect is noticeable after the appropriate selection of stimulation parameters, especially the time period of the application of the vibration exercise, the frequency of the applied vibration, and perhaps the number of series of vibrations per session. In this study, data demonstrated that younger women showed larger improvements in posturographic parameters after SR-WBV. Other authors showed [25–30] a positive influence of SR-WBV on the ability to maintain balance in the elderly and those with various neurological diseases, so the weaker effects achieved in older women compared to the younger ones in this study are rather surprising. Ditrich et al. [25], Hartmann et al. [26], Turbanski et al. [27], and Priplata et al. [28, 30] did not focus on age and did not analyse the influence of this factor on the effectiveness of SR-WBV training. However, Priplata et al. [29] found that greater benefits from vibration were derived among elderly rather than among younger people. In their research, a completely different generating method was used (vibrating insoles under the feet), the vibrations had different characteristics (frequency 100 Hz), and other methods were used to assess postural stability. Therefore, the results are not comparable. A study that directly compares the effectiveness of both study methods is necessary to demonstrate which process should be recommend for older people.

A progressive atrophy of the skeletal muscles, and decrease in the ability to regenerate muscle fibres, which consequently impairs the functioning of the nervous system is observed during the ageing process. This might be a cause of the lower efficiency of vibration in elderly women, as presented in this study [36]. To achieve results comparable to those seen in younger people, the modification of SR-WBV parameters or total duration of exercise may be necessary. While analysing the influence of age on SR-WBV training effects, Rogan and Radlinger [37] results are worth to be mentioned. The results showed that the most important aspect in the selection of training is the current physical performance level and not the age. When planning next studies this aspect is worth considering.

In the present study, we demonstrated that taller participants and those with lower BMIs displayed better effects of SR-WBV. Data collected by the Central Statistical Office [38] regarding the growth of the adult Polish population suggest that younger people generally are taller than elderly people. Statistically, the average height of a 20-year old Polish woman is 166 cm, so she is 3 cm higher than her mother, and 5 cm than her grandmother. In the present study, a similar correlation between age and height was observed. Therefore, lower participant age may explain the greater efficiency of SR-WBV.

Blaszczyk [39] evaluated the effect of body weight on stability and found that higher BMIs resulted in smaller COP excursions in the ML direction, while in a free-standing position. Reduced reactivity among obese participants was observed only during position changes or balance disturbances. In obese women, fat accumulates mainly in the lower half of the body, especially the hip and thigh areas. In addition, when in a free standing position, their legs are wider apart from each other, which significantly reduces medial-lateral excursions. Therefore, we can conclude that the lower index of improvement of posturographic parameters are the results of lower output parameters, associated with the movement of the centre of gravity of the body line.

Limitations of this study are the selection of the study group and the selection of the training program. In the aims of this study the effect of SR-WBV training on the ability to maintain balance and the usefulness of SR-WBV in falls prevention were supposed to be studied. The answer to this question could be achieved by selecting participants with imbalances and falls in their medical history and elderly participants from the high risk of falls group. These individuals, however, suffer from many accompanying diseases. The research group had had little experience in using the SR-WBV so it was decided to begin the study with young and healthy participants using the strongest rehabilitation program. Such a stimulus and group selection make the results unclear as to whether the SR-WBV training using the muscle imbalance program would benefit the target group who have a medical history of falls. Considering the deterioration in the health of the four women with either no or only a minor health problem in their medical history, the conclusion might be drawn that the stimuli were too strong for the majority of those older people classified as being at high risk of having a fall. The positive results obtained in young healthy women provide two pieces of practical information: the muscle imbalance program is useful in such individuals and further study is needed to find the most optimal vibration parameters for the elderly and for people with a health problem in their medical history.

It is worth mentioning that there was no control group in this study. While analysing factors that could affect the balance beyond the influence of stochastic resonance vibrations, the following are worth mentioning: the seasonal changes in physical activity (summer weather in Poland favors activity increase), additional physical activity connected with actually getting to the training room and the effect of the position with slightly bent knees 9 times during 45 seconds with 45 seconds pause during the training session. The seasonal changes of activity should not have a significant impact on results, because the study was conducted throughout the whole year, with the exception of the summer months. Physically getting to the training sessions for students, employees or the University of the Third Age attendees should not be a sufficient stimulus to effect changes in balance. A forced standing position with bent knees is the factor that could most affect balance.

One of the limitations of this work is the use of the parameters in falls prevention while a predictive ability has not been confirmed in prospective studies. Although literature [7, 9, 10, 11] shows that posturographic parameters describing the movement of COP along the X-axis are strong indicators of the stability and probability of falls, these are not prospective studies. Swanenberg et. al [40] have shown that the only posturographic parameter that effectively predicts falls in the following year is the root mean square amplitude in medial–lateral directions (RMS-ML). The software offered by the manufacturer of double-plate posturography used in this study does not calculate this parameter. This is the reason why the mean velocity and the mean amplitude of the movement of COP along the x-axis (medial-lateral) and the general mean velocity of the movement of COP were chosen, although their ability to predict falls has not been confirmed. In further studies, it would be good to improve the software and to add supplementary parameters to remove this limitation.

Another limitation of this study is the lack of reliability/validity of the measurement approach.

It would also be beneficial to use functional tests such as ETGUG: Expanded Timed Get-Up-and-Go, where, after SR-WBV training, statistically significant improvement was found in pilot studies, in contrast to posturographic measurements where no effect was observed [35].

## Conclusions

As a result of SR-WBV stimulation, the left leg is more stable and the disproportion between the stability of both legs is reduced. Consequently, body stability in a standing position is higher. The SR-WBV training is more effective in younger, taller and slimmer females. Training parameters should be optimized so that the training is more beneficial for elderly and shorter women and for women with a higher BMI.

## Supporting information

S1 Table Additional posturographic parameters measured in female volunteers during the test with eyes open, pre and post SR-WBV. SP—the sway path, SPAP—the sway path along the y-axis, SPML—the sway path along the x-axis, MA—the mean amplitude, MAAP—the mean amplitude along the y-axis, MVAP—the mean velocity along the y-axis, SA—the sway area, MF—the mean frequency, TR—the time radius, EO- the test performed by volunteers with eyes open, L- the parameters calculated independently for the left lower limb, R- the parameters calculated independently for the right lower limb, B- the resultant parameters calculated for both limbs, pre SR-WBV- the test performed before SR-WBV training, post SR-WBV- the test performed after SR-WBV, x—mean, sd—standard deviation, p—significance, PHP—post-hoc power, ns—not significant.
(PDF)

S2 Table Additional posturographic parameters measured in female volunteers during the test with eyes closed, pre and post SR-WBV. SP—the sway path, SPAP—the sway path along the y-axis, SPML—the sway path along the x-axis, MA—the mean amplitude, MAAP—the mean amplitude along the y-axis, MVAP—the mean velocity along the y-axis, SA—the sway area, MF—the mean frequency, TR—the time radius, EC- the test performed in volunteers with eyes closed, L- the parameters calculated independently for the left lower limb, R- the parameters calculated independently for the right lower limb, B- the resultant parameters calculated for both limbs, pre SR-WBV- the test performed before SR-WBV training, post SR-WBV- the test performed after SR-WBV training, x—mean, sd—standard deviation, p—significance, PHP—post-hoc power, ns—not significant.
(PDF)

S3 Table Disproportion of the values of additional posturographic parameters of lower limbs pre and post SR-WBV. IR-LI—the module of different values obtained for the right and left lower limbs, SP—the sway path, SPAP—the sway path along the y-axis, SPML—the sway path along the x-axis, MA—the mean amplitude, MAAP—the mean amplitude along the y-axis, MVAP—the mean velocity along the y-axis, SA—the sway area, MF—the mean frequency, TR—the time radius, EO- the test performed by volunteers with eyes open, EC- the test performed by volunteers with eyes closed, pre SR-WBV- the test performed before SR-WBV trainings, post SR-WBV- the test performed after SR-WBV trainings, x—mean, sd—standard deviation, p—significance, PHP—post-hoc power, ns—not significant.
(PDF)

S4 Table Correlations between age, height, BMI and the index of improvement of additional posturographic parameters in female volunteers during the test with eyes open. SP—the sway path, SPAP—the sway path along the y-axis, SPML—the sway path along the x-axis, MA—the mean amplitude, MAAP—the mean amplitude along the y-axis, MVAP—the mean velocity along the y-axis, SA—the sway area, MF—the mean frequency, TR—the time radius, EO- the test performed by volunteers with eyes open, L- the parameters calculated independently for the left lower limb, R- the parameters calculated independently for the right lower limb, B- the parameters calculated as the resultant of both limbs, r = rank correlation values, p—significance, ns—not significant.
(PDF)

S5 Table Correlations between age, height, BMI and the index of improvement of additional posturographic parameters in female volunteers during the test with eyes closed. SP—the sway path, SPAP—the sway path along the y-axis, SPML—the sway path along the x-axis, MA—the mean amplitude, MAAP—the mean amplitude along the y-axis, MVAP—the mean velocity along the y-axis, SA—the sway area, MF—the mean frequency, TR—the time radius, EC- the test performed by volunteers with eyes closed, L- the parameters calculated independently for the left lower limb, R- the parameters calculated independently for the right lower limb, B- the resultant parameters calculated for both limbs, r = rank correlation values, p—significance, ns—not significant.
(PDF)
