# Supplementary material for: Alterations in the ability to maintain balance as a result of stochastic resonance whole body vibration in women
Source: PLoS One. 2017 Sep 22;12(9):e0185179. doi: 10.1371/journal.pone.0185179 (PMC5609760; doi:10.1371/journal.pone.0185179)
Supplement: S1 Table — SP—the sway path, SPAP—the sway path along the y-axis, SPML—the sway path along the x-axis, MA—the mean amplitude, MAAP—the mean amplitude along the y-axis, MVAP—the mean velocity along the y-axis, SA—the sway area, MF—the mean frequency, TR—the time radius, EO- the test performed by volunteers with eyes open, L- the parameters calculated independently for the left lower limb, R- the parameters calculated independently for the right lower limb, B- the resultant parameters calculated for both limbs, pre SR-WBV- the test performed before SR-WBV training, post SR-WBV- the test performed after SR-WBV, x—mean, sd—standard deviation, p—significance, PHP—post-hoc power, ns—not significant. (PDF) [file pone.0185179.s001.pdf]

Parameter	SR-WBV	x	sd	p /PHP	Parameter	SR-WBV	x	sd	p /PHP	Parameter	SR-WBV	x	sd	p
**SP-EO-B [mm]**	**pre**	**203.2**	**57.6**	**=0.07 /0.3**	**SP-EO-L [mm]**	pre	213.8	48.6	ns	**SP-EO-R [mm]**	pre	211.4	65.7	ns
	**post**	**197.6**	**44.4**			post	208.2	48.4			post	206.6	47.5	
**SPAP-EO-B [mm]**	pre	139.6	41.9	ns	**SPAP-EO-L [mm]**	pre	163.3	41.6	ns	**SPAP-EO-R [mm]**	pre	171.0	64.1	ns
	post	136.2	34.2			post	160.4	43.2			post	165.3	46.2	
**SPML-EO-B [mm]**	pre	116.6	39.4	ns	**SPML-EO-L [mm]**	**pre**	**107.8**	**24.4**	**<0.01 /0.7**	**SPML-EO-R [mm]**	pre	93.5	19.7	ns
	post	113.3	29.3			**post**	**103.4**	**21.1**			post	94.2	16.7	
**MA-EO-B [mm]**	pre	3.04	1.34	ns	**MA-EO-L [mm]**	pre	2.83	1.77	ns	**MA-EO-R [mm]**	pre	3.07	1.40	ns
	post	3.16	1.34			post	2.79	1.51			post	3.26	1.57	
**MAAP-EO-B [mm]**	pre	2.57	1.32	ns	**MAAP-EO-L [mm]**	pre	2.64	1.74	ns	**MAAP-EO-R [mm]**	pre	2.90	1.40	ns
	post	2.70	1.30			post	2.63	1.50			post	3.10	1.56	
**MVAP-EO-B [mm/s]**	pre	4.65	1.40	ns	**MVAP-EO-L [mm/s]**	pre	5.44	1.39	ns	**MVAP-EO-R [mm/s]**	pre	5.70	2.13	ns
	post	4.54	1.14			post	5.35	1.44			post	5.51	1.54	
**SA-EO-B [mm^2]**	pre	192.2	125.7	ns	**SA-EO-L [mm^2]**	pre	155.1	116.0	ns	**SA-EO-R [mm^2]**	pre	143.4	78.9	ns
	post	195.2	107.5			post	146.3	90.5			post	151.9	80.7	
**MF-EO-B [Hz]**	pre	0.41	0.17	ns	**MF-EO-L [Hz]**	pre	0.51	0.26	ns	**MF-EO-R [Hz]**	pre	0.43	0.19	ns
	post	0.38	0.15			post	0.50	0.27			post	0.41	0.18	
**TR-EO -B [%]**	pre	84.3	17.2	ns	**TR-EO-L [%]**	pre	84.8	19.0	ns	**TR-EO-R [%]**	pre	81.7	17.7	ns
	post	82.2	18.4			post	85.0	18.7			post	78.9	20.3	
