# Supplementary material for: Alterations in the ability to maintain balance as a result of stochastic resonance whole body vibration in women
Source: PLoS One. 2017 Sep 22;12(9):e0185179. doi: 10.1371/journal.pone.0185179 (PMC5609760; doi:10.1371/journal.pone.0185179)
Supplement: S2 Table — SP—the sway path, SPAP—the sway path along the y-axis, SPML—the sway path along the x-axis, MA—the mean amplitude, MAAP—the mean amplitude along the y-axis, MVAP—the mean velocity along the y-axis, SA—the sway area, MF—the mean frequency, TR—the time radius, EC- the test performed in volunteers with eyes closed, L- the parameters calculated independently for the left lower limb, R- the parameters calculated independently for the right lower limb, B- the resultant parameters calculated for both limbs, pre SR-WBV- the test performed before SR-WBV training, post SR-WBV- the test performed after SR-WBV training, x—mean, sd—standard deviation, p—significance, PHP—post-hoc power, ns—not significant. (PDF) [file pone.0185179.s002.pdf]

Parameter	SR-WBV	x	sd	p /PHP	Parameter	SR-WBV	x	sd	p /PHP	Parameter	SR-WBV	x	sd	p /PHP
**SP-EC-B [mm]**	**pre**	**235.9**	**75.5**	**<0.05 /0.6**	**SP-EC-L [mm]**	pre	239.8	62.6	ns	**SP-EC-R [mm]**	**pre**	**253.6**	**97.0**	**<0.05 /0.7**
	**post**	**226.2**	**61.7**			post	236.6	66.9			**post**	**238.8**	**70.6**	
**SPAP-EC-B [mm]**	pre	177.2	65.7	ns	**SPAP-EC-L [mm]**	pre	190.6	58.8	ns	**SPAP-EC-R [mm]**	**pre**	**214.0**	**95.2**	**<0.01 /0.8**
	post	170.7	56.7			post	190.3	65.1			**post**	**198.6**	**71.6**	
**SPML-EC-B [mm]**	**pre**	**117.4**	**40.2**	**<0.05 /0.7**	**SPML-EC-L [mm]**	**pre**	**112.2**	**24.4**	**<0.01 /0.9**	**SPML-EC-R [mm]**	pre	100.7	29.7	ns
	**post**	**112.0**	**29.2**			**post**	**107.3**	**22.6**			post	98.7	20.0	
**MA-EC-B [mm]**	pre	2.75	1.32	ns	**MA-EC-L [mm]**	pre	2.51	1.55	ns	**MA-EC-R [mm]**	pre	2.91	1.43	ns
	post	2.78	1.23			post	2.54	1.39			post	2.97	1.42	
**MAAP-EC-B [mm]**	pre	2.41	1.30	ns	**MAAP-EC-L [mm]**	pre	2.34	1.54	ns	**MAAP-EC-R [mm]**	pre	2.76	1.39	ns
	post	2.46	1.19			post	2.40	1.38			post	2.82	1.42	
**MVAP-EC-B [mm/s]**	pre	5.91	2.19	ns	**MVAP-EC-L [mm/s]**	pre	6.35	1.96	ns	**MVAP-EC-R [mm/s]**	**pre**	**7.13**	**3.17**	**<0.01 /0.7**
	post	5.69	1.89			post	6.34	2.17			**post**	**6.62**	**2.39**	
**SA-EC-B [mm^2]**	pre	187.9	129.8	ns	**SA-EC-L [mm^2]**	pre	142.4	98.4	ns	**SA-EC-R [mm^2]**	pre	143.2	82.4	ns
	post	185.0	117.9			post	138.3	90.4			post	144.3	78.1	
**MF-EC-B [Hz]**	**pre**	**0.51**	**0.18**	**<0.05 /0.6**	**MF-EC-L [Hz]**	pre	0.62	0.26	ns	**MF-EC-R [Hz]**	pre	0.52	0.19	ns
	**post**	**0.48**	**0.17**			post	0.60	0.30			post	0.50	0.20	
**TR-EC-B [%]**	pre	87.0	16.0	ns	**TR-EC-L [%]**	pre	87.8	16.3	ns	**TR-EC-R [%]**	pre	83.3	17.2	ns
	post	86.8	15.0			post	87.7	15.0			post	82.9	16.6	
