# Supplementary material for: Alterations in the ability to maintain balance as a result of stochastic resonance whole body vibration in women
Source: PLoS One. 2017 Sep 22;12(9):e0185179. doi: 10.1371/journal.pone.0185179 (PMC5609760; doi:10.1371/journal.pone.0185179)
Supplement: S3 Table — IR-LI—the module of different values obtained for the right and left lower limbs, SP—the sway path, SPAP—the sway path along the y-axis, SPML—the sway path along the x-axis, MA—the mean amplitude, MAAP—the mean amplitude along the y-axis, MVAP—the mean velocity along the y-axis, SA—the sway area, MF—the mean frequency, TR—the time radius, EO- the test performed by volunteers with eyes open, EC- the test performed by volunteers with eyes closed, pre SR-WBV- the test performed before SR-WBV trainings, post SR-WBV- the test performed after SR-WBV trainings, x—mean, sd—standard deviation, p—significance, PHP—post-hoc power, ns—not significant. (PDF) [file pone.0185179.s003.pdf]

Parameter	SR-WBV	x	sd	p /PHP	Parameter	SR-WBV	x	sd	p /PHP
**IR-LI SP-EO [mm]**	pre	44.1	41.3	ns	**IR-LI SP-EC [mm]**	pre	62.2	60.2	ns
	post	40.3	30.9			post	53.8	49.0	
**IR-LI SPAP-EO [mm]**	pre	43.3	43.6	ns	**IR-LI SPAP-EC [mm]**	pre	62.3	62.9	ns
	post	39.1	31.9			post	56.2	50.8	
**IR-LI SPML-EO [mm]**	**pre**	**21.6**	**17.1**	**<0.05 /0.7**	**IR-LI SPML-EC [mm]**	**pre**	**23.9**	**20.7**	**<0.01 /0.7**
	**post**	**17.9**	**12.7**			**post**	**19.5**	**14.5**	
**IR-LI MA-EO [mm]**	pre	1.22	1.27	ns	**IR-LI MA-EC [mm]**	pre	1.05	0.93	ns
	post	1.20	1.12			post	1.06	0.95	
**IR-LI MAAP-EO [mm]**	pre	1.22	1.27	ns	**IR-LI MAAP-EC [mm]**	pre	1.03	0.95	ns
	post	1.21	1.11			post	1.07	0.95	
**IR-LI MVAP-EO [mm/s]**	pre	1.44	1.45	ns	**IR-LI MVAP-EC [mm/s]**	pre	2.08	2.10	ns
	post	1.30	1.07			post	1.87	1.69	
**IR-LI SA-EO [mm^2]**	pre	69.0	84.4	ns	**IR-LI SA-EC [mm^2]**	pre	64.3	64.4	ns
	post	64.6	60.7			post	58.5	57.3	
**IR-LI MF-EO [Hz]**	pre	0.18	0.17	ns	**IR-LI MF-EC [Hz]**	pre	0.18	0.16	ns
	post	0.17	0.20			post	0.19	0.21	
**IR-LI TR-EO [%]**	pre	14.5	14.4	ns	**IR-LI TR-EC [%]**	pre	11.8	11.1	ns
	post	14.4	15.4			post	11.9	11.2	
