# Supplementary material for: Alterations in the ability to maintain balance as a result of stochastic resonance whole body vibration in women
Source: PLoS One. 2017 Sep 22;12(9):e0185179. doi: 10.1371/journal.pone.0185179 (PMC5609760; doi:10.1371/journal.pone.0185179)
Supplement: S4 Table — SP—the sway path, SPAP—the sway path along the y-axis, SPML—the sway path along the x-axis, MA—the mean amplitude, MAAP—the mean amplitude along the y-axis, MVAP—the mean velocity along the y-axis, SA—the sway area, MF—the mean frequency, TR—the time radius, EO- the test performed by volunteers with eyes open, L- the parameters calculated independently for the left lower limb, R- the parameters calculated independently for the right lower limb, B- the parameters calculated as the resultant of both limbs, r = rank correlation values, p—significance, ns—not significant. (PDF) [file pone.0185179.s004.pdf]

	Parameter	r	p	Parameter	r	p	Parameter	r	p
**Age/ Index of improvement**	SP-EO-B [mm]	-0,04	ns	SP-EO-L [mm]	-0,11	ns	SP-EO-P [mm]	0,06	ns
	SPAP-EO-B [mm]	-0,08	ns	SPAP-EO-L [mm]	-0,13	ns	SPAP-EO-P [mm]	0,07	ns
	SPML-EO-B [mm]	0,06	ns	SPML-EO-L [mm]	0,00	ns	SPML-EO-P [mm]	-0,04	ns
	MA-EO-B [mm]	0,05	ns	MA-EO-L [mm]	-0,01	ns	MA-EO-P [mm]	0,06	ns
	MAAP-EO-B [mm]	0,03	ns	MAAP-EO-L [mm]	0,00	ns	MAAP-EO-P [mm]	0,08	ns
	MVAP-EO-B [mm/s]	-0,09	ns	MVAP-EO-L [mm/s]	-0,14	ns	MVAP-EO-P [mm/s]	0,07	ns
	SA-EO-B [mm^2]	0,05	ns	SA-EO-L [mm^2]	-0,02	ns	SA-EO-P [mm^2]	0,06	ns
	MF-EO-B [Hz]	-0,09	ns	MF-EO-L [Hz]	-0,02	ns	MF-EO-P [Hz]	-0,06	ns
	TR-EO-B [%]	-0,05	ns	TR-EO-L [%]	-0,05	ns	TR-EO-P [%]	-0,06	ns
**Height/ Index of improvement**	SP-EO-B [mm]	0,07	ns	SP-EO-L [mm]	0,09	ns	SP-EO-P [mm]	0,02	ns
	SPAP-EO-B [mm]	0,12	ns	SPAP-EO-L [mm]	0,09	ns	SPAP-EO-P [mm]	0,02	ns
	SPML-EO-B [mm]	0,00	ns	SPML-EO-L [mm]	0,03	ns	SPML-EO-P [mm]	0,04	ns
	MA-EO-B [mm]	0,04	ns	MA-EO-L [mm]	0,10	ns	MA-EO-P [mm]	-0,02	ns
	MAAP-EO-B [mm]	0,04	ns	MAAP-EO-L [mm]	0,09	ns	MAAP-EO-P [mm]	-0,03	ns
	MVAP-EO-B [mm/s]	0,12	ns	MVAP-EO-L [mm/s]	0,10	ns	MVAP-EO-P [mm/s]	0,02	ns
	SA-EO-B [mm^2]	0,02	ns	SA-EO-L [mm^2]	0,09	ns	SA-EO-P [mm^2]	-0,02	ns
	MF-EO-B [Hz]	-0,07	ns	MF-EO-L [Hz]	-0,06	ns	MF-EO-P [Hz]	0,02	ns
	TR-EO-B [%]	0,00	ns	TR-EO-L [%]	-0,11	ns	TR-EO-P [%]	0,02	ns
**BMI/ Index of improvement**	SP-EO-B [mm]	-0,02	ns	SP-EO-L [mm]	0,00	ns	SP-EO-P [mm]	0,03	ns
	SPAP-EO-B [mm]	-0,01	ns	SPAP-EO-L [mm]	0,01	ns	SPAP-EO-P [mm]	0,05	ns
	SPML-EO-B [mm]	-0,04	ns	SPML-EO-L [mm]	0,01	ns	SPML-EO-P [mm]	-0,04	ns
	MA-EO-B [mm]	0,11	ns	MA-EO-L [mm]	0,06	ns	MA-EO-P [mm]	0,10	ns
	MAAP-EO-B [mm]	0,11	ns	MAAP-EO-L [mm]	0,07	ns	MAAP-EO-P [mm]	0,09	ns
	MVAP-EO-B [mm/s]	-0,02	ns	MVAP-EO-L [mm/s]	0,01	ns	MVAP-EO-P [mm/s]	0,05	ns
	SA-EO-B [mm^2]	0,08	ns	SA-EO-L [mm^2]	0,04	ns	SA-EO-P [mm^2]	0,09	ns
	MF-EO-B [Hz]	-0,13	ns	MF-EO-L [Hz]	-0,06	ns	MF-EO-P [Hz]	-0,10	ns
	TR-EO-B [%]	-0,13	ns	TR-EO-L [%]	-0,08	ns	TR-EO-P [%]	-0,07	ns
