# Supplementary material for: Alterations in the ability to maintain balance as a result of stochastic resonance whole body vibration in women
Source: PLoS One. 2017 Sep 22;12(9):e0185179. doi: 10.1371/journal.pone.0185179 (PMC5609760; doi:10.1371/journal.pone.0185179)
Supplement: S5 Table — SP—the sway path, SPAP—the sway path along the y-axis, SPML—the sway path along the x-axis, MA—the mean amplitude, MAAP—the mean amplitude along the y-axis, MVAP—the mean velocity along the y-axis, SA—the sway area, MF—the mean frequency, TR—the time radius, EC- the test performed by volunteers with eyes closed, L- the parameters calculated independently for the left lower limb, R- the parameters calculated independently for the right lower limb, B- the resultant parameters calculated for both limbs, r = rank correlation values, p—significance, ns—not significant. (PDF) [file pone.0185179.s005.pdf]

	Parameter	r	p	Parameter	r	p	Parameter	r	p
**Age/ Index of improvement**	**SP-EC-B [mm]**	**-0,23**	**<0,01**	**SP-EC-L [mm]**	**-0,29**	**<0,001**	SP-EC-P [mm]	-0,06	ns
	**SPAP-EC-B [mm]**	**-0,24**	**<0,01**	**SPAP-EC-L [mm]**	**-0,29**	**<0,001**	SPAP-EC-P [mm]	-0,06	ns
	SPML-EC-B [mm]	-0,06	ns	**SPML-EC-L [mm]**	**-0,19**	**<0,05**	SPML-EC-P [mm]	-0,09	ns
	MA-EC-B [mm]	-0,08	ns	MA-EC-L [mm]	-0,13	ns	MA-EC-P [mm]	0,01	ns
	MAAP-EC-B [mm]	-0,06	ns	MAAP-EC-L [mm]	-0,14	ns	MAAP-EC-P [mm]	0,00	ns
	**MVAP-EC-B [mm/s]**	**-0,24**	**<0,01**	**MVAP-EC-L [mm/s]**	**-0,29**	**<0,001**	MVAP-EC-P [mm/s]	-0,06	ns
	SA-EC-B [mm^2]	-0,14	ns	**SA-EC-L [mm^2]**	**-0,17**	**<0,05**	SA-EC-P [mm^2]	-0,02	ns
	MF-EC-B [Hz]	-0,07	ns	MF-EC-L [Hz]	0,01	ns	MF-EC-P [Hz]	-0,07	ns
	TR-EC-B [%]	0,07	ns	TR-EC-L [%]	0,12	ns	TR-EC-P [%]	-0,04	ns
**Height/ Index of improvement**	SP-EC-B [mm]	0,06	ns	**SP-EC-L [mm]**	**0,23**	**<0,01**	SP-EC-P [mm]	-0,07	ns
	SPAP-EC-B [mm]	0,08	ns	**SPAP-EC-L [mm]**	**0,20**	**<0,01**	SPAP-EC-P [mm]	-0,06	ns
	SPML-EC-B [mm]	-0,04	ns	**SPML-EC-L [mm]**	**0,15**	**<0,05**	SPML-EC-P [mm]	-0,07	ns
	MA-EC-B [mm]	0,00	ns	MA-EC-L [mm]	0,05	ns	MA-EC-P [mm]	-0,06	ns
	MAAP-EC-B [mm]	-0,03	ns	MAAP-EC-L [mm]	0,03	ns	MAAP-EC-P [mm]	-0,04	ns
	MVAP-EC-B [mm/s]	0,08	ns	**MVAP-EC-L [mm/s]**	**0,20**	**<0,01**	MVAP-EC-P [mm/s]	-0,06	ns
	SA-EC-B [mm^2]	0,05	ns	SA-EC-L [mm^2]	0,12	ns	SA-EC-P [mm^2]	-0,07	ns
	MF-EC-B [Hz]	0,03	ns	MF-EC-L [Hz]	0,04	ns	MF-EC-P [Hz]	0,02	ns
	TR-EC-B [%]	-0,03	ns	TR-EC-L [%]	-0,06	ns	TR-EC-P [%]	0,08	ns
**BMI/ Index of improvement**	**SP-EC-B [mm]**	**-0,16**	**<0,05**	SP-EC-L [mm]	-0,11	ns	SP-EC-P [mm]	-0,04	ns
	SPAP-EC-B [mm]	-0,10	ns	SPAP-EC-L [mm]	-0,09	ns	SPAP-EC-P [mm]	-0,03	ns
	**SPML-EC-B [mm]**	**-0,16**	**<0,05**	SPML-EC-L [mm]	-0,04	ns	SPML-EC-P [mm]	-0,03	ns
	MA-EC-B [mm]	-0,02	ns	MA-EC-L [mm]	-0,08	ns	MA-EC-P [mm]	0,06	ns
	MAAP-EC-B [mm]	0,00	ns	MAAP-EC-L [mm]	-0,08	ns	MAAP-EC-P [mm]	0,06	ns
	MVAP-EC-B [mm/s]	-0,11	ns	MVAP-EC-L [mm/s]	-0,09	ns	MVAP-EC-P [mm/s]	-0,03	ns
	SA-EC-B [mm^2]	-0,09	ns	SA-EC-L [mm^2]	-0,10	ns	SA-EC-P [mm^2]	0,06	ns
	MF-EC-B [Hz]	-0,06	ns	MF-EC-L [Hz]	0,06	ns	MF-EC-P [Hz]	-0,12	ns
	TR-EC-B [%]	0,00	ns	TR-EC-L [%]	0,04	ns	TR-EC-P [%]	-0,04	ns
